# Development and Validation of an LC-MS/MS Based Method for the Determination of Deoxynivalenol and Its Modified Forms in Maize

**DOI:** 10.3390/toxins13090600

**Published:** 2021-08-27

**Authors:** Iris Fiby, Marta Magdalena Sopel, Herbert Michlmayr, Gerhard Adam, Franz Berthiller

**Affiliations:** 1 Institute of Bioanalytics and Agro-Metabolomics, Department of Agrobiotechnology (IFA-Tulln), University of Natural Resources and Life Sciences, Vienna (BOKU), Konrad Lorenz Str. 20, 3430 Tulln, Austria; iris.fiby@gmail.com (I.F.); marta_magdalena@onet.eu (M.M.S.); 2 Institute of Microbial Genetics, Department of Applied Genetics and Cell Biology, BOKU, Konrad Lorenz Str. 24, 3430 Tulln, Austria; herbert.michlmayr@boku.ac.at (H.M.); gerhard.adam@boku.ac.at (G.A.)

**Keywords:** mycotoxins, trichothecenes, masked mycotoxins, modified mycotoxins, mass spectrometry, stable-isotope dilution assay

## Abstract

The Fusarium mycotoxin deoxynivalenol (DON) is a common contaminant of cereals and is often co-occurring with its modified forms DON-3-glucoside (D3G), 3-acetyl-DON (3ADON) or 15-acetyl-DON (15ADON). A stable-isotope dilution liquid chromatography-tandem mass spectrometry (LC-MS/MS) based method for their determination in cereals was developed and validated for maize. Therefore, ^13^C-labelled D3G was enzymatically produced using ^13^C-DON and [^13^C_6_Glc]-sucrose and used as an internal standard (IS) for D3G, while uniformly ^13^C labelled IS was used for the other mycotoxins. Baseline separation was achieved for the critical peak pair DON/D3G, while 3ADON/15ADON could not be fully baseline separated after testing various reversed phase, fluorinated phase and chiral LC columns. After grinding, weighing and extracting the cereal samples, the raw extract was centrifuged and a mixture of the four ^13^C-labelled ISs was added directly in a microinsert vial. The subsequent analytical run took 7 min, followed by negative electrospray ionization and selected reaction monitoring on a triple quadrupole MS. Maize was used as a complex cereal model matrix for validation. The use of the IS corrected the occurring matrix effects efficiently from 76 to 98% for D3G, from 86 to 103% for DON, from 68 to 100% for 15ADON and from 63 to 96% for 3ADON.

## 1. Introduction

Mycotoxins are low molecular weight, secondary metabolites of fungi of different genera, which may cause serious health implications for mammals, when ingested with food or feed, as reviewed by [1]. One of the most prevalent groups of mycotoxins—trichothecenes—contains a tetracyclic sesquiterpenoid 12,13-epoxytrichothec-9-en ring structure, with the epoxide group responsible for the typical trichothecenes’ toxic effects, as reviewed by [2]. Trichothecenes are produced by plant pathogenic *Fusarium* spp., growing preferably on cereals in the field at temperate climates [3]. The type B trichothecene deoxynivalenol (DON) is one of the most commonly found mycotoxins worldwide [4]. Its toxic effects include emesis (hence, its colloquial name “vomitoxin”), anorexia, growth retardation, immunotoxicity, impaired reproduction and development, altered neuroendocrine signaling, proinflammatory gene induction and altered gut integrity [5].

**Figure 1:**
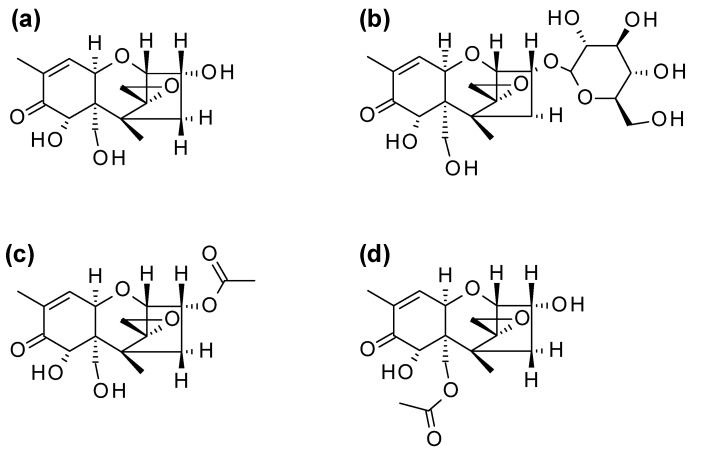
Chemical structures of (**a**) deoxynivalenol (DON) and its modified forms (**b**) deoxynivalenol-3-glucoside (D3G), (**c**) 3-acetyldeoxynivalenol (3ADON) and (**d**) 15-acetyldeoxynivalenol (15ADON).

DON is often co-occurring with its acetylated biosynthetical precursors 3-acetyl-DON (3ADON) or 15-acetyl-DON (15ADON), and its plant metabolite DON-3-glucoside (D3G, Figure 1) in cereals, such as wheat, barley, oats, rye and maize or products thereof (e.g., [6,7]). As both the acetylated and the glucosidic forms of DON can be easily hydrolyzed to DON in vivo [8], the toxicity of those so-called “modified mycotoxins” [9] is basically the same as that of the DON for humans. As such, the European Food Safety Authority proposed a group tolerable daily intake value of 1 µg/kg bodyweight for the sum of the four compounds [10].

The most popular technique to determine mycotoxins in food nowadays [11,12] is liquid chromatography coupled to mass spectrometry (LC-MS). Typically reversed phase chromatography on a C-18 column is used to separate mycotoxins of different polarities, before they are charged during electrospray ionization and subsequently analyzed with MS [13]. LC-MS offers tremendous sensitivity, selectivity and multiplexing capability, but accurate quantification is often challenged due to matrix effects [14]. As such, co-eluting matrix compounds suppress or enhance the signal (SSE), compared to standards in neat solvents (external calibration). One of the most sophisticated ways to cope with matrix effects is the usage of stable isotope labeled internal standards. Those have the same physico-chemical properties as the analytes, but different molecular masses and do not occur in nature. Several stable isotope dilution assays (SIDA) have been developed for accurate mycotoxin determination so far, including the most important mycotoxins (e.g., [15,16,17]). While uniformly labeled ^13^C-DON, ^13^C-3ADON and ^13^C-15ADON are commercially available, until very recently, ^13^C-D3G was not. Habler et al. proposed the Königs–Knorr method to chemically synthesize DON-3-[^13^C_6_]-glucoside from unlabeled DON and [^13^C_6_]-labeled glucose. The authors successfully applied the compound as IS for the analysis of beer samples for the concurrent determination of DON and D3G [18].

Another challenge in mass spectrometry is the determination of isomers, as they—by definition—share the same molecular formula and mass. If specific MS/MS fragmentation is unavailable, chromatographic separation should be aimed for. In the case of 3ADON and 15ADON, this separation is hard to achieve and in many multi-toxin methods, those two compounds co-elute (e.g., [19]). While the loss of a CH_2_O group at C-15 during collision induced dissociation in MS/MS allows the formation of a specific fragment of 3ADON in negative ion mode (*m*/*z* 307), no such specific ion is available for 15ADON, severely limiting its quantification.

The major aim of this work was to develop and validate a robust, fast and accurate LC-MS/MS based method that allows the concurrent determination of DON along with its major modified forms 3ADON, 15ADON and D3G. We enzymatically produced and purified uniformly labeled ^13^C-D3G and used it together with ^13^C-DON, ^13^C-3ADON and ^13^C-15ADON as internal standards. Chromatographic separation was optimized, allowing near base-line separation of 3ADON and 15ADON. To the best of our knowledge, this is the first stable-isotope dilution assay covering those four mycotoxins.

## 2. Results

### 2.1. MS Method Optimization

One of the main prerequisites to developing a SIDA method was the production of ^13^C-D3G, which was commercialized later. U-[^13^C_21_]-D3G was synthesized in a batch conversion containing 100 mg ^13^C-DON. The batch contained 21 mM U-[^13^C_15_]-DON, 32 mM [^13^C_6_Glc]-sucrose (β-D-fructofuranosyl-α-D-[U-^13^C_6_]glucopyranoside; 99 atom% ^13^C, Omicron Biochemicals Inc., South Bend, IN, USA), 1 mM UDP, 100 mM potassium phosphate pH 7. UDP-glucosyltransferase OsUGT79 and sucrose synthase AtSUS1 [20] were added at 1.5 mg/mL each. The reaction was incubated at 37 °C. After 48 hrs, the batch contained less than 0.5% un-conjugated DON. U-[^13^C_21_]-D3G was isolated by preparative HPLC (Agilent 1100 series, Waldbronn, Germany) and freeze-dried. The total yield was 76%.

**Figure 2:**
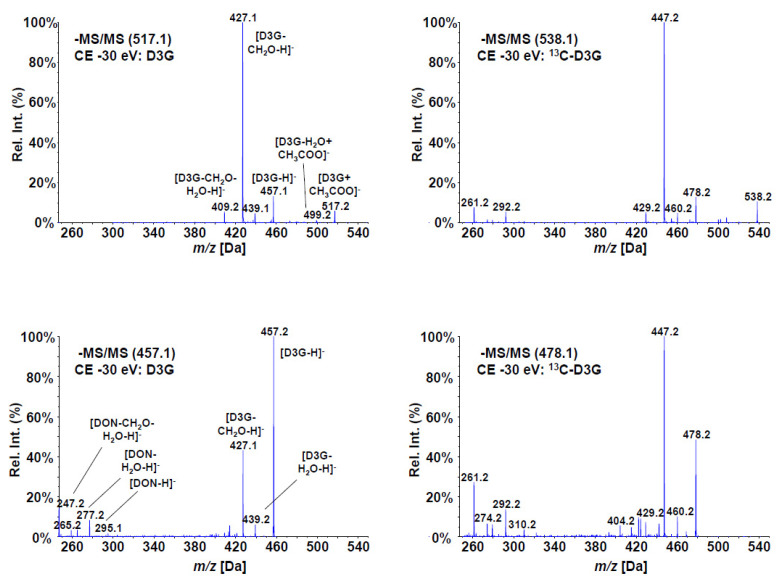
Enhanced Product Ion (MS/MS) spectra of D3G (left) and ^13^C-D3G (right) at a collision energy of 30 eV. The spectra derived from the acetate adducts are shown on top, the spectra using the deprotonated precursors on the bottom.

The electrospray (ESI) MS/MS fragmentation spectra of the produced compound, as well as those of unlabeled D3G at 30 eV collision energy, are shown in Figure 2. All major fragments of D3G were found with ^13^C-D3G with the according mass shifts (+21 amu for the precursor due the sum formula of C_21_H_30_O_11_). One of the main fragments is the loss of a CH_2_O group from the C-15 backbone of DON (*m*/*z* 427 or 447 for the labeled compound). Despite the same collision energy, that fragment was more abundant than the deprotonated precursor of ^13^C-D3G, but less abundant using the deprotonated D3G as precursor for reasons unknown. However, the acetate adduct resulted in much higher overall intensities and the fragmentation pattern of D3G and of ^13^C-D3G were virtually identical.

Consecutive syringe pump optimization was performed for all analytes and IS for both the acetate adducts and the deprotonated precursors and used for method development. After coupling with LC, it could be seen that the acetate adducts of all analytes gave higher signal to noise ratios; hence, three transitions for each analyte—always using the [M+CH_3_COO]^−^ precursors—were selected for the final method (Table 1). The entrance potentials were kept at 10 V for each transition. Dwell times of 20 ms and pause times of 5 ms between transitions resulted in a cycle time of 0.45 s.

### 2.2. LC Method Optimization

Different types of chromatography (reversed phased, chiral) and 13 different columns (including C18 and pentafluorophenyl phases) were evaluated to separate the critical peak pairs of DON/D3G and 3ADON/15ADON. Ammonium acetate (2 mM) was added into both mobile phases to ensure acetate ion adduct formation in negative ESI mode. With all conditions, acetonitrile (ACN) gave better separation for 3ADON/15ADON than methanol (MeOH) and was therefore chosen as the organic solvent for the final method. For DON/D3G, separation was similar for both mobile phases. The retention times and resolutions of the critical peak pairs for the tested columns, using the ACN mobile phase, are summarized in Table 2.

**Figure 3:**
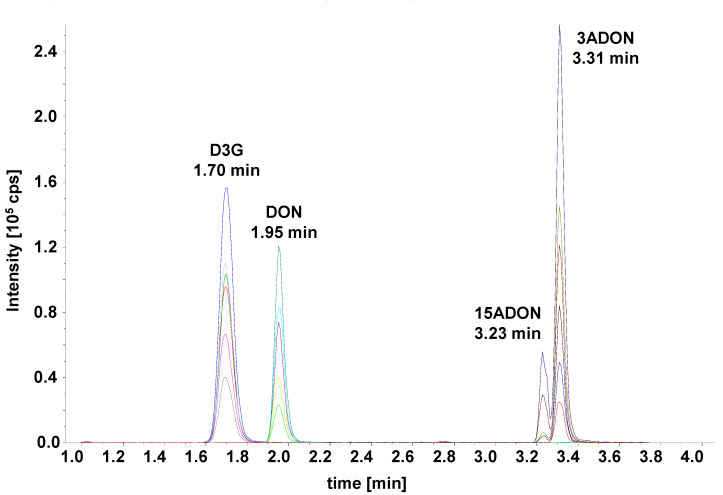
LC-MS/MS selected ion monitoring (SRM) chromatogram of the optimized method. Separation was achieved on a Waters Acquity UPLC HSS T3 C18, 1.8 µm, 2.1 × 100 mm column using a water–acetonitrile gradient.

Only three columns (marked bold in Table 2) yielded resolutions ≥2.0 for both peak pairs. Of those, the Waters Acquity HSS T3 yielded the highest separation power and was used for further optimization. The gradient was optimized and the run time shortened to develop the final method (see chromatogram in Figure 3).

### 2.3. Method Validation

Prior to method validation, different extraction solvents (20%, 50%, 80% aqueous ACN) were tested. All three solvents yielded almost identical apparent recoveries (R_A_). As expected, the matrix effects (SSE) were slightly less pronounced with more apolar solvents, while the extraction recoveries (R_E_) were slightly higher with more polar solvents (data not shown). As the matrix effects are supposed to be corrected by the IS, ACN/H_2_O, 20/80 (*v*/*v*) was chosen as extraction solvent. In addition, this yielded the advantage that the raw solvent can be directly injected into the LC-MS/MS system without causing peak distortion.

The determined method performance parameters for maize include apparent recovery (R_A_), matrix effects (SSE) and extraction recovery (R_E_) and are summarized in Table 3. For limits of quantification (LOQ) determination, the spiked maize samples were used which consistently yielded signal to noise ratios exceeding 10. Linearity has been shown for neat standard solutions in the range of 3–1000 µg/L (equaling 12–4000 µg/kg) with squared linear calibration coefficients exceeding 0.998 for all analytes.

## 3. Discussion

Our aim was to develop a robust yet accurate, fast and easy to use LC-MS/MS based method for the determination of DON and its major metabolites for routine food analysis. In order to do so, it was first imperative to produce and characterize ^13^C-D3G, which is now available to all stakeholders. The applied enzymatic strategy [20] can be easily scaled up and yielded uniformly labelled ^13^C_21_-D3G for the first time, already using ^13^C_15_-DON [15] as starting compound.

Another—often ignored—issue in mycotoxin determination was partly solved with the accurate quantification of 3ADON and 15ADON. The two compounds can be easily separated using gas chromatography [21], but barely using LC even by applying long run-times [22]. *Fusarium* spp. originally produce 3,15-diacetyl-DON that in the final step of biosynthesis is deacetylated by an esterase encoded by the TRI8 gene [2]. Different alleles of this gene determine production of either 3ADON or 15ADON chemotypes in *F. graminearum*. While often samples are only contaminated with one chemotype, e.g., complex food samples or mixed feedstuffs easily can contain both acetylated forms. For a toxicological point of view, it is important to differ between the compounds, as there are some differences in gastro-intestinal deleterious effects and relative toxicity, with 15ADON often being the more potent toxin [8]. From an analytical-chemical point of view, it is relevant to observe differences in ionization of the compounds (see Figure 3), so a sum value with either or both toxins, as standards will lead to inaccurate quantification when there is no chromatographical separation. Multi-mycotoxin methods based on LC-MS/MS are widely used nowadays [11,12], but remain a compromise in various aspects in order to “squeeze in” a multitude of analytes in a single method. In case accurate quantification of 3ADON and 15ADON is warranted, methods not resolving the compounds cannot be recommended. Due to the specific fragmentation of 3ADON, this compound can be accurately determined even in the presence of 15ADON with such methods, e.g., [19]. While this proposed method does not fully solve the issue, the separation of 3ADON and 15ADON is sufficient to accurately quantify them. While we used the peak areas for the quantification (due to only low crosstalk from the overlapping peaks), a suitable option would be to use the peak heights instead (after using the same peak smoothing settings for all samples and standards).

Several different extraction solvents were tested in this study. In (multi-analyte) mycotoxin determination, a very common solvent is ACN/H_2_O/acetic acid (79/20/1, *v*/*v*/*v*) [19]. Acidification is important for the extraction of several (charged) mycotoxins, but not for type B-trichothecenes, e.g., [21,22]. The high ratio of organic solvent usually serves multiple purposes: (a) more apolar mycotoxins than type B-trichothecenes can be extracted, (b) the solvent is compatible with several clean-up strategies and (c) fewer polar matrix compounds are co-extracted, yielding fewer matrix effects for polar analytes. Considering compensation of matrix effects by the used ISs, we opted for the solvent composition with the highest extraction recoveries, which was ACN/H_2_O, 20/80 (*v*/*v*). Choosing this solvent also allows direct injection in the UHPLC system after the addition of ISs and avoids a further dry-down step.

More sensitive methods for the determination of type B-trichothecenes are available in literature, recently reviewed in [23]. Such methods often need a clean-up and concentration step, which could be avoided here. Sample preparation is not only faster and cheaper (even considering the costs of the ISs), but less error-prone and more robust. This was made possible by the use of a highly sensitive MS system. In case such a system is unavailable, up-concentration is a requirement to achieve suitable limits of quantification. Mass spectrometric performance varies from day to day, and in the worst case, also during measurement. The use of standards before, after and in the middle of the sequence is therefore recommended. We did notice only a negligible decrease of the slopes of the individual calibration curves during a batch, allowing us to use all replicates for the evaluation of the results. We did, however, notice day-to-day variations in sensitivity, as the instrument is used by multiple users and running different methods (and matrices). As such, we refrained from the determination of limits of detections, which is often performed under optimal system performance. Instead, we used standards with concentrations of 3, 10, 30, 100 µg/L and defined the LOQ for that level which always showed signal/noise ratios higher than 10. This approach resulted in LOQs of 10 µg/L (40 µg/kg) for all analytes, but the rather poorly ionizing 15ADON (LOQ 30 µg/L or 120 µg/kg). With maximum regulated levels of 200–1750 µg/kg for DON in Europe [24], we consider the method sensitive enough for routine applications. In case low background levels are the study subject, an intermediate up-concentration step would be required.

On purpose, we added the ISs only after extraction, only compensating matrix effects during measurements and random injection volume variations. The use of conical micro-inserts allows minimizing the amount of ISs, thus saving costs. Unconventionally, we also opted to simply dilute 80 µL of raw extracts with 20 µL of ISs in the vials—resulting in a dilution factor for the samples. However, this factor is offset if the standards are prepared in the same manner. For example, 80 µL of a 100 µg/L neat standard solution are diluted with 20 µL of ISs, but further regarded as 100 µg/L standard (despite its actual concentration of only 80 µg/L). Using this little trick, no back calculations (other than the dilution factor of four for extraction) are needed for data evaluation.

It is sometimes believed that the repeatability of a method could always be improved using ISs. In our case, which is in agreement with current literature, e.g., [16,17], this was not the case. Already excellent RSDr values of 3–9% for all analytes using external calibration changed to 5–8% using internal calibration. Expected interlaboratory reproducibilies RSD_R_ of 32.0%, 22.6% and 16.0% for levels of 10, 100 and 1000 µg/kg, according to [25], are likely to be reached. Thus, even further improvement of the method precision by using IS was unlikely and not obtained.

Concluding, the presented method offers a robust manner to accurately determine DON and its major metabolites in cereals, using minimal sample preparation if a highly sensitive LC-MS/MS system is available.

## 4. Materials and Methods

### 4.1. Chemicals

Acetonitrile (ACN, gradient grade) was purchased from VWR International GmbH (Vienna, Austria), methanol (MeOH, ≥99.9 %) was obtained from Honeywell (Seelze, Germany), ammonium acetate (LC-MS grade) was provided by Sigma Aldrich (Vienna, Austria). Ultrapure water was produced by an ELGA Purelab Ultra system (Celle, Germany). All standards were provided by Romer Labs GmbH (Tulln, Austria). The individual stock standard solutions (all in ACN) had the following concentrations: DON 100.5 µg/mL, 3ADON 100.4 µg/mL, 15ADON 100.1 µg/mL, D3G 50.4 µg/mL, U-[^13^C_15_]-DON 25.1 µg/mL (99.0 atom% ^13^C), U-[^13^C_17_]-3ADON 25.2 µg/mL (99.4 atom% ^13^C), U-[^13^C_17_]-15ADON 10.0 µg/mL (99.1% ^13^C). U-[^13^C_21_]-D3G became recently available from Romer Labs (10.6 µg/mL, 99.2 atom% ^13^C).

### 4.2. Samples

Different maize samples (1 kg each) used for the method development were bought at a health food store in Tulln, Austria, and milled with a Romer Analytical Sampling Mill from Romer Labs GmbH (Getzersdorf, Austria). For the determination of natural mycotoxin contamination, samples were extracted and measured with a multi-mycotoxin method [19]. A popcorn maize from controlled organic cultivation (Rapunzel Naturkost, Germany) was shown to be uncontaminated with 3ADON, 15ADON and D3G and was used as a blank for method validation. DON was only found in traces, below the LOQ of the used method (<10 µg/kg).

### 4.3. LC-MS/MS Optimization

All LC-MS/MS measurements were performed on a 1290 series ultra-high performance liquid chromatography system (Agilent Technologies, Waldbronn, Germany) coupled to a QTrap 6500+ MS/MS System (Sciex, Foster City, CA, USA) equipped with a IonDrive Turbo V electrospray ionization (ESI) source. Analysis was carried out using the dynamic selective reaction monitoring mode (SRM) with monitoring of two transitions (quantifier and qualifier).

Precursor and product ion selection as well as the optimization of declustering potentials (DP), entrance potentials (EP), collision energies (CE) and cell exit potentials (CXP) were performed with flow injection of single analyte solutions of 1 mg/L concentration using a Hamilton syringe and the Analyst 1.6.3. software in negative mode (ESI-). The source temperature was 550 °C. From each analyte, the acetate adduct and the deprotonated adduct were scanned.

For the optimization of the separation of DON, D3G, 3ADON and 15ADON, a 1 mg/L working solution in ACN/H_2_O, 40/60, *v*/*v* was used. Different UHPLC columns, of C18, perfluorinated and chiral materials were tested for this purpose using two following LC methods, where eluent A was composed of 5% MeOH (or ACN) and eluent B of 98% MeOH (or ACN), both containing 2 mM ammonium acetate. Chromatographic separation was performed at 25 °C with a flow rate of 0.4 mL/min for columns with a length of 50 mm, and a flow rate of 0.2 mL/min for columns with a length of 100 or 150 mm. The injection volume was set to 3 μL. The total chromatographic run time was 15 min. Different gradients’ initial conditions (10, 20 and 30% of B) and slopes with an intermediate step at (20, 30, 40 and 50% B) were tested prior to shortening the method from 15 to 7 min. Tested columns were purchased from Agilent Technologies (Waldbronn, Germany), Daicel (Chiral Technologies Europe SAS, Illkirch-Graffenstaden, France), Phenomenex (Aschaffenburg, Germany), Sigma-Aldrich, ThermoFisher Scientific (Vienna, Austria) or Waters (Vienna, Austria) and are shown in Table 4.

As DON/D3G as well as 3ADON/15ADON yielded nearly identical peak width, the resolution was calculated with the following equation.

(1) $$R_{S}=\frac{t2-t1}{w}$$

Unconventionally, we used the peak at the half maximum (FWHM) rather than the peak at its baseline for the calculations. The reason for that was to minimize the influence of slightly differently integrated peaks (thus, a potentially differently selected baseline) for the selection of the best-suited stationary phase.

Finally, the chromatographic separation was performed on a Waters Acquity UPLC HSS T3 C18, 1.8 µm, 2.1 × 100 mm column at 25 °C with a flow rate of 0.4 mL/min and 3 µL injection volume. Eluent A was composed of 5% ACN and eluent B of 98% ACN, both containing 2 mM ammonium acetate. The chromatographic separation of the analytes was achieved in a total run time of 7 min, with a gradient comprising an initial hold time of 0.5 min at 10% B and a linear gradient to 44% B within 3 min. The gradient was set to 100% B afterwards to wash the column till 5.4 min, followed to re-equilibration at 10% B until the end of the run. A switching valve directed the LC flow to the MS from 1.0 to 3.8 min.

### 4.4. Method Validation

Recovery experiments were performed by spiking blank maize samples (1.00 ± 0.01 g) with the appropriate amount of spiking solution of unlabeled mycotoxins at six levels (resulting in expected measurement values of 3, 10, 30, 100, 300 and 1000 µg/L) in triplicate before extraction. The concentrations of spiking solutions used were 10.0 mg/L, 1.00 mg/L and 100 µg/L of DON, D3G, 3ADON and 15ADON solved in pure ACN. Fifteen mL polypropylene tubes with spiked samples were allowed to rest in the hood overnight at room temperature to allow solvent evaporation and to achieve equilibrium between the analytes and matrix. On the next day, the samples were extracted with 4.00 mL ACN/H_2_O, 20/80 (*v*/*v*) for 60 min on a shaker at room temperature and centrifuged (3500 rpm). Subsequently, 20 µL of IS working solution consisting of a 500 µg/L concentration of ^13^C-DON, ^13^C-D3G, ^13^C-3ADON and ^13^C-15ADON dissolved in ACN/H_2_O, 20:80, *v/v*, was added to 80 µL of the supernatant in an HPLC vial fitted with a 200 µL conical glass insert.

To evaluate matrix effects, blank maize samples (5.00 ± 0.01 g) were extracted in triplicates with 20 mL of ACN/H_2_O, 20/80 (*v*/*v*) for 60 min on a rotary shaker (200 rpm) at room temperature and centrifuged (3500 rpm). Matrix-matched standards were prepared at six levels (+a blank level) in triplicates. For this purpose, working standard solutions of mycotoxins were pipetted into HPLC vials, evaporated and reconstituted with 500 µL of raw extract. This resulted in a spiking level of 0, 3.00, 10.0, 30.0, 100, 300 and 1000 µg/L. Afterwards, 80 μL of these solutions were mixed with 20 μL IS working solution in an HPLC vial containing a microinsert.

Sample preparation in general consisted of cereal extraction with the four-fold volume of ACN/H_2_O, 20/80 (*v*/*v*) for 60 min on a rotary shaker, followed by centrifugation. Always, 80 µL of either raw extracts or standard solutions in neat solvents were diluted with 20 µL IS solution (500 µg/L of all four labelled compounds) directly in the HPLC microinsert prior to analysis.

### 4.5. Data Evaluation

For data evaluation, 1/× weighted calibration curves were obtained for each analyte by plotting the relative response versus the analyte concentration using Analyst 1.6.3 (Sciex, Concord, ON, Canada) software. The peak area of the analyte divided by the peak area of the corresponding internal standard was the relative response. The analytes concentrations were calculated by the relative response and the calibration curves with internal calibration. Apparent recoveries were calculated by the ratio of measured to spiked concentrations, followed by calculating the average value of all six spiking levels in triplicate analysis, expressed in percent.

For the evaluation of matrix effects, the data were first analyzed without considering the internal standards, which led to the determination of the apparent recoveries for external calibration. Furthermore, signal suppression or enhancement (SSE) of the SIDA method was calculated from the spiked blank extracts in the same way. To calculate the extraction recovery (R_E_), mean values of the apparent recovery using internal calibration (R_A_) were divided by the mean values of the signal suppression or enhancement (SSE). The repeatability (RSD_r_) was calculated from the triplicate analysis at seven spiking levels.

## Figures and Tables

**Table 1:** List of analytes with optimized ESI-MS/MS parameters.

Analyte ID	Q1 Mass (Da)	Q3 Mass (Da)	DP (V)	CE (eV)	CXP (V)
D3G 1	517.1	457.1	−80	−22	−7
D3G IS 1	538.1	478.1	−80	−22	−7
D3G 2	517.1	59.0	−80	−74	−9
D3G IS 2	538.1	59.0	−80	−74	−9
D3G 3	517.1	427.0	−80	−32	−5
D3G IS 3	538.1	447.0	−80	−32	−5
DON 1	355.0	59.0	−70	−36	−9
DON IS 1	370.0	59.0	−70	−36	−9
DON 2	355.0	295.0	−70	−16	−13
DON IS 2	370.0	310.0	−70	−16	−13
DON 3	355.0	265.0	−70	−24	−13
DON IS 3	370.0	279.0	−70	−24	−13
ADONs 1	397.1	59.0	−70	−34	−9
ADON IS 1	414.1	49.0	−70	−34	−9
ADONs 2	397.1	337.1	−70	−12	−7
ADON IS 2	414.1	354.1	−70	−12	−7
3ADON	397.1	307.0	−70	−22	−5
3ADON IS	414.1	323.0	−70	−22	−5

D3G deoxynivalenol-3-glucoside, DON deoxynivalenol, ADONs acetyl-deoxynivalenols, 3ADON 3-acetyl-deoxynivalenol, IS internal standard, DP declustering potential, CE collision energy, CXP cell exit potential.

**Table 2:** Evaluated analytical columns for the LC-MS/MS method.

Column	t_R_ (DON) (min)	t_R_ (D3G) (min)	t_R_ (3ADON) (min)	t_R_ (15ADON) (min)	FWHM (min)	R_S_ (DON/D3G)	R_S_ (3/15ADON)
Agilent Zorbax Eclipse Plus	2.63	2.68	4.16	4.17	0.045	−1.11	−0.22
Agilent Zorbax Extend-C18	2.15	2.26	3.85	3.86	0.077	−1.43	−0.13
**Agilent Poroshell EC-C18**	**5.30**	**5.14**	**7.30**	**7.16**	**0.057**	**2.82**	**2.47**
Agilent Zorbax SB C18	5.87	5.87	9.00	9.02	0.085	0.00	−0.24
**Agilent Zorbax XDB-C18**	**4.75**	**4.52**	**6.58**	**6.46**	**0.060**	**3.83**	**2.00**
Daicel Chiralcel	3.59	3.45	11.00	9.00	3.000	0.05	0.67
Daicel Chiralpak	2.29	2.06	9.29	9.08	0.143	1.60	1.47
Phenomenex Kinetex C18	4.07	4.03	5.81	5.82	0.075	0.53	−0.13
Phenomenex Kinetex F5	4.26	4.15	6.33	6.23	0.067	1.65	1.50
Sigma Discovery HS F5	6.14	5.95	9.88	9.68	0.122	1.56	1.64
Thermo Hypersil Gold	1.84	2.13	3.68	3.69	0.112	−2.60	−0.09
Waters Acquity BEH C18	2.98	3.21	4.37	4.40	0.040	−5.75	−0.75
**Waters Acquity HSS T3 C18**	**5.20**	**4.93**	**7.17**	**7.02**	**0.052**	**5.23**	**2.90**

t_R_ retention time, FWHM full width half maximum, Rs resolution. A water–acetonitrile gradient (5% → 60% acetonitrile in 9 min) containing 2 mM ammonium acetate was used for separation. The flow rate was 0.2 or 0.4 mL/min, depending on the column length.

**Table 3:** Method performance parameters.

Analyte	LOQ Solution (μg/L)	LOQ Maize (μg/kg)	R_E_ (%)	SSE (%)	R_A_ (%)	RSDr (%)
D3G (ext.)	<10	<40	94.7	75.9	92.3	3.1
D3G (int.)				97.5		8.3
DON (ext.)	<10	<40	101	86.4	104	5.9
DON (int.)				103		5.3
15ADON (ext.)	<30	<120	105	67.8	105	9.4
15ADON (int.)				100		7.8
3ADON (ext.)	<10	<40	94.4	63.2	90.8	7.0
3ADON (int.)				96.2		5.7

LOQ: limit of quantification; R_E_: extraction recovery; SSE: signal suppression or enhancement; R_A_: apparent recovery; RSDr: relative standard deviation under conditions of repeatability; ext. external calibration; int. internal calibration.

**Table 4:** Evaluated analytical columns for the LC-MS/MS method.

Supplier	Brand Name	Dimensions (mm)	Particle Size (µm)
Agilent	ZORBAX RRHD Eclipse Plus C18	2.1 × 50	1.8
Agilent	ZORBAX RRHT Extend-C18	2.1 × 50	1.8
Thermo	Hypersil GOLD C18	2.1 × 50	1.9
Waters	ACQUITY UPLC BEH C18	2.1 × 50	1.7
Agilent	ZORBAX RRHD StableBond C18	2.1 × 100	1.8
Agilent	ZORBAX RRHD Eclipse XDB-C18	2.1 × 100	1.8
Phenomenex	Kinetex C18	2.1 × 100	2.6
Waters	ACQUITY UPLC HSS T3 (C18)	2.1 × 100	1.8
Agilent	InfinityLab Poroshell 120 EC-C18	2.1 × 150	2.7
Phenomenex	Kinetex F5	2.1 × 100	2.6
Sigma-Aldrich	Discovery HS F5	2.1 × 100	5.0
Daicel	CHIRALPAK AD-3R	2.1 × 150	3.0
Daicel	CHIRALCEL OJ-3R	2.1 × 150	3.0

## References

[1] Alshannaq A., Yu J.-H. (2017). Occurrence, Toxicity, and Analysis of Major Mycotoxins in Food. Int. J. Environ. Res. Public Health.

[2] McCormick S.P., Stanley A.M., Stover N.A., Alexander N.J. (2011). Trichothecenes: From Simple to Complex Mycotoxins. Toxins.

[3] Ma L.-J., Geiser D.M., Proctor R.H., Rooney A.P., O’Donnell K., Trail F., Gardiner D.M., Manners J.M., Kazan K. (2013). FusariumPathogenomics. Annu. Rev. Microbiol..

[4] Khaneghah A.M., Fakhri Y., Raeisi S., Armoon B., Sant’Ana A.S. (2018). Prevalence and concentration of ochratoxin A, zearalenone, deoxynivalenol and total aflatoxin in cereal-based products: A systematic review and meta-analysis. Food Chem. Toxicol..

[5] Pestka J.J. (2010). Deoxynivalenol: Mechanisms of action, human exposure, and toxicological relevance. Arch. Toxicol..

[6] Berthiller F., Dall’Asta C., Corradini R., Marchelli R., Sulyok M., Krska R., Adam G., Schuhmacher R. (2009). Occurrence of deoxynivalenol and its 3-β-D-glucoside in wheat and maize. Food Addit. Contam. Part A.

[7] Varga E., Malachova A., Schwartz H., Krska R., Berthiller F. (2013). Survey of deoxynivalenol and its conjugates deoxynivalenol-3-glucoside and 3-acetyl-deoxynivalenol in 374 beer samples. Food Addit. Contam. Part A.

[8] Payros D., Alassane-Kpembi I., Pierron A., Loiseau N., Pinton P., Oswald I.P. (2016). Toxicology of deoxynivalenol and its acetylated and modified forms. Arch. Toxicol..

[9] Rychlik M., Humpf H.-U., Marko D., Dänicke S., Mally A., Berthiller F., Klaffke H., Lorenz N. (2014). Proposal of a comprehensive definition of modified and other forms of mycotoxins including “masked” mycotoxins. Mycotoxin Res..

[10] EFSA Panel on Contaminants in the Food Chain (2017). Risks to human and animal health related to the presence of deoxynivalenol and its acetylated and modified forms in food and feed. EFSA J..

[11] Tittlemier S., Cramer B., Dall’Asta C., Iha M., Lattanzio V., Maragos C., Solfrizzo M., Stranska M., Stroka J., Sumarah M. (2020). Developments in mycotoxin analysis: An update for 2018–2019. World Mycotoxin J..

[12] Tittlemier S., Brunkhorst J., Cramer B., DeRosa M., Lattanzio V., Malone R., Maragos C., Stranska M., Sumarah M. (2021). Developments in mycotoxin analysis: An update for 2019–2020. World Mycotoxin J..

[13] Malachová A., Stránská M., Václavíková M., Elliott C.T., Black C., Meneely J., Hajslova J., Ezekiel C.N., Schuhmacher R., Krska R. (2018). Advanced LC–MS-based methods to study the co-occurrence and metabolization of multiple mycotoxins in cereals and cereal-based food. Anal. Bioanal. Chem..

[14] Li P., Zhang Z., Hu X., Zhang Q. (2013). Advanced hyphenated chromatographic-mass spectrometry in mycotoxin determination: Current status and prospects. Mass Spectrom. Rev..

[15] Häubl G., Berthiller F., Krska R., Schuhmacher R. (2006). Suitability of a fully 13C isotope labeled internal standard for the determination of the mycotoxin deoxynivalenol by LC-MS/MS without clean up. Anal. Bioanal. Chem..

[16] Varga E., Glauner T., Köppen R., Mayer K., Sulyok M., Schuhmacher R., Krska R., Berthiller F. (2012). Stable isotope dilution assay for the accurate determination of mycotoxins in maize by UHPLC-MS/MS. Anal. Bioanal. Chem..

[17] Habler K., Rychlik M. (2015). Multi-mycotoxin stable isotope dilution LC-MS/MS method for Fusarium toxins in cereals. Anal. Bioanal. Chem..

[18] Habler K., Frank O., Rychlik M. (2016). Chemical Synthesis of Deoxynivalenol-3-β-D-[13C_6_]-glucoside and Application in Stable Isotope Dilution Assays. Molecules.

[19] Malachová A., Sulyok M., Beltran E., Berthiller F., Krska R. (2014). Optimization and validation of a quantitative liquid chromatography–tandem mass spectrometric method covering 295 bacterial and fungal metabolites including all regulated mycotoxins in four model food matrices. J. Chromatogr. A.

[20] Michlmayr H., Malachová A., Varga E., Kleinová J., Lemmens M., Newmister S., Rayment I., Berthiller F., Adam G. (2015). Biochemical Characterization of a Recombinant UDP-glucosyltransferase from Rice and Enzymatic Production of Deoxynivalenol-3-O-β-d-glucoside. Toxins.

[21] Josephs R., Krska R., Grasserbauer M., Broekaert J. (1998). Determination of trichothecene mycotoxins in wheat by use of supercritical fluid extraction and high-performance liquid chromatography with diode array detection or gas chromatography with electron capture detection. J. Chromatogr. A.

[22] Buttinger G., Krska R. (2003). Determination of B-trichothecenes in wheat by post column derivatisation liquid chromatography with fluorescence detection (PCD-HPLC-FLD). Mycotoxin Res..

[23] Polak-Śliwińska M., Paszczyk B. (2021). Trichothecenes in Food and Feed, Relevance to Human and Animal Health and Methods of Detection: A Systematic Review. Molecules.

[24] European Commission (2006). Commission Regulation (EC) No 1881/2006 of 19 December 2006. Setting maximum levels for certain contaminants in foodstuffs. Off. J. Eur. Union.

[25] Horwitz W. (1982). Evaluation of Analytical Methods Used for Regulation of Foods and Drugs. Anal. Chem..

